# Novel chemistry of invasive plants: exotic species have more unique metabolomic profiles than native congeners

**DOI:** 10.1002/ece3.1132

**Published:** 2014-06-14

**Authors:** Mirka Macel, Ric C H de Vos, Jeroen J Jansen, Wim H van der Putten, Nicole M van Dam

**Affiliations:** 1 Department of Terrestrial Ecology, Netherlands Institute of Ecology (NIOO-KNAW)P.O. Box 50, 6700 AB, Wageningen, The Netherlands; 2 Molecular Interaction Ecology, Institute of Water and Wetland Research (IWWR), Radboud University NijmegenP.O. Box 9010, 6500 GL, Nijmegen, The Netherlands; 3 Plant Ecology, University of TübingenAuf der Morgenstelle 3, 72076, Tübingen, Germany; 4 Plant Research International, Wageningen University and Research Centre (WUR)P.O. Box 17, 6700 AA, Wageningen, The Netherlands; 5 Centre for BioSystems and GenomicsP.O. box 98, 6700 AB, Wageningen, The Netherlands; 6 Department of Analytical Chemistry, Radboud University NijmegenHeyendaalseweg 135, 6525 AJ, Nijmegen, The Netherlands; 7 Laboratory of Nematology, Wageningen University and Research Centre (WUR)P.O. Box 8123, 6700 ES, Wageningen, The Netherlands

**Keywords:** Asteraceae, herbivory, LC-MS, *Mamestra brassicae*, metabolomics, novel weapons, range expanding, secondary metabolites, *Senecio*, *Solidago*

## Abstract

It is often assumed that exotic plants can become invasive when they possess novel secondary chemistry compared with native plants in the introduced range. Using untargeted metabolomic fingerprinting, we compared a broad range of metabolites of six successful exotic plant species and their native congeners of the family Asteraceae. Our results showed that plant chemistry is highly species-specific and diverse among both exotic and native species. Nonetheless, the exotic species had on average a higher total number of metabolites and more species-unique metabolites compared with their native congeners. Herbivory led to an overall increase in metabolites in all plant species. Generalist herbivore performance was lower on most of the exotic species compared with the native species. We conclude that high chemical diversity and large phytochemical uniqueness of the exotic species could be indicative of biological invasion potential.

## Introduction

Many plant species have been introduced to other continents either accidentally or by deliberate introduction for, for example, horticultural purposes. Moreover, over the past few decades, distributions of species have shifted pole-wards and will continue to do so under current and future climate change (Parmesan and Yohe 2003; Walther 2010). The redistribution of species and changing climatic conditions can lead to biological invasions, whereby exotic species increasingly dominate native ecosystems and alter various aspects of ecosystem functioning (Thuiller et al. 2007; Vila et al. 2011).

There are many different hypotheses on why exotic plants may become invasive (Catford et al. 2009). The enemy release hypothesis (Keane and Crawley 2002) assumes that the loss of specialist natural enemies in the new range releases the plants from top-down control and contributes to biological invasions. The evolution of increased competitive ability (EICA) hypothesis (Blossey and Notzold 1995) predicts this leads to a decrease in chemical defenses and increased competitive ability. The novel weapons hypothesis, on the other hand, poses that exotic plants may have secondary compounds in their root exudates that are not found in native plants, which are toxic to unadapted native species (Callaway and Aschehoug 2000; Callaway and Ridenour 2004). This hypothesis can be extended to shoot chemistry and its effect on aboveground unadapted native herbivores or pathogens (Cappuccino and Arnason 2006; Barto et al. 2010; Schaffner et al. 2011; Enge et al. 2012). A literature review revealed that invasive exotic plants are indeed more likely to have unique secondary compounds that are not found in noninvasive exotic plants and native plants, suggesting that novel chemistry can indeed contribute to invasion success (Cappuccino and Arnason 2006). Most results cited in this review were based on chemical analyses targeted toward specific (groups of) known defense compounds. Thus far, experimental studies investigating the role of novel plant chemistry in biological invasions focused on one or a few compound classes and/or one or a few plant species (Callaway et al. 2008; Lankau et al. 2009; Barto et al. 2010; Enge et al. 2012; Kaur et al. 2012; Whitehill et al. 2012; Qin et al. 2013; Svensson et al. 2013). One of the more broad studies to date analyzed several phenolic compounds of nine native and nine invasive plant species (Kim and Lee 2011). However, comprehensive chemical analytical techniques, for example, untargeted metabolomics, nowadays enable the simultaneous screening of several hundreds of known and unknown plant metabolites belonging to different chemical classes that may be present in a single plant species (Fiehn et al. 2000; Macel et al. 2010). Such an untargeted metabolomic profiling or fingerprinting approach provides a much broader view of plant chemistry. Moreover, because of the global extraction and analysis approach, it can be applied to any plant species. Metabolomic profiling has, for example, been used to identify previously unknown plant defense compounds in *Chrysanthum* (Leiss et al. 2009) and *Brassica oleracea* (Jansen et al. 2009).

Here, we used a comprehensive untargeted metabolomics approach to investigate the differences in shoot chemistry of a range of successful range-expanding exotic plant species and their native sister species of the same genus. We also tested the performance of a native generalist herbivore (*Mamestra brassicae* L.) on the plants, and analyzed the effect of herbivore damage on the metabolomics profiles. We expected that the exotic species would be phytochemically unique, with compounds not found in the native plants (Cappuccino and Arnason 2006) and that herbivore performance would be lower on the exotic species. We also expected the chemical profiles to change slightly after herbivore attack due to the possible induction of defenses (Karban and Baldwin 1997). By investigating both uninduced and herbivory-induced plants, we could cover a wider range of the metabolome of the plants. Most studies on exotic plant defenses only considered constitutive defense levels (but see Cipollini et al. 2005).

All selected plant species were of the Asteraceae family because we expected the chemical defenses within one plant family to be more comparable than between families. Three of the exotic species, *Senecio inaequidens*, *Solidago gigantea*, and *Bidens frondosa* were introduced in Europe from other continents and are among the most invasive terrestrial plants in Western Europe (Lambdon et al. 2008). *Se. inaequidens* is known to contain moderate amounts of (hepato) toxic pyrrolizidine alkaloids, which are also found in native *Senecio* species (Cano et al. 2009). Native snails readily fed on the exotic *Senecio* species, while a native specialist herbivore that is adapted to the alkaloids did not survive on it (Macel et al. 2002; Cano et al. 2009). The latter suggests that other compounds besides pyrrolizidine alkaloids are playing a role in herbivore resistance in the invasive *Senecio* species. *So. gigantea* is known to contain various commonly occurring terpenoids (Hull-Sanders et al. 2009) and the chemistry of *B. frondosa* is largely unknown thus far. The other three exotic species, *Artemisia biennis*, *Tragopogon dubius*, and *Tanacetum parthenium* are Eurasian plants native to South or South East Europe. They are exotic in North West Europe where they have been increasing in abundance over the last 50 years (Tamis et al. 2005). An earlier study found that these range-expanding plants are less affected by herbivores, possibly due to higher total levels of phenolic compounds (Engelkes et al. 2008). Similar to essential oils of their native congeners, extracts of both *A. biennis* and *Ta. parthenium* contain a rich diversity of terpenoids that may have antibiotic or insecticidal properties (Lopes-Lutz et al. 2008; Wolf et al. 2011). Other than this, little is known about the defense chemistry of these range-expanding exotic species. Plants were grown in the greenhouse and received either no herbivory or herbivory by the generalist herbivore *M. brassicae*. LC-MS metabolomics on the shoots was performed and larval weight of *M. brassicae* before and after feeding on the plants was measured.

## Material and Methods

### Plant and herbivore species

For each exotic plant species, we chose a native relative from the same genus co-occurring with the exotics in the invaded habitat (Table 1), so we could make a phylogenetically controlled comparison. Not all the exotic species are considered highly invasive but they all have been increasing in abundance in the Netherlands over the last 50 years (Tamis 2005). Three exotic plant species originated from other continents, whereas three other exotic species were intracontinental range expanders within Eurasia. All plants were grown from seed collected from wild local Dutch populations by Dutch seed companies. Larvae of the cabbage moth, *Mamestra brassicae* (Lepidoptera; Noctuidae), were obtained from a laboratory rearing at the Entomology Department of Wageningen University, the Netherlands where they were reared on cabbage for many generations. This native palearctic generalist herbivore feeds from plant of many different families, including the Asteraceae (Theunissen et al. 1985). We used third instars, reared on artificial diet, in the experiment.

**Table 1:** Origin of species used in the experiment

Plant species	Origin	Dutch population used in experiment	Present since
*Artemisia biennis*	Eurasia	Dodewaard	1950
*Artemisia vulgaris*	Eurasia	Gendtse Polder	Native
*Bidens frondosa*	North America	Polder Zeevang	1900
*Bidens tripartita*	Eurasia	Polder Zeevang	Native
*Senecio inaequidens*	South Africa	Millingerwaard	1925
*Senecio vulgaris*	Eurasia	Heerlen	Native
*Senecio jacobaea*	Eurasia	Millingerwaard/Meijendel	Native
*Solidago gigantea*	North America	Gendtse Polder	1900
*Solidago virgaurea*	Eurasia	Seed company	Native
*Tanacetum parthenium*	Eurasia	Seed company	1500
*Tanacetum vulgare*	Eurasia	Seed company	Native
*Tragopogon dubius*	Eurasia	Amersfoort	1925
*Tragopogon pratensis*	Eurasia	Ooijpolder	Native

Underlined species names indicate exotic species. Some exotic plants originate in Eurasia and are non-native to the Netherlands, others originate from other continents.

### Experiment

Seeds were surface sterilized with a 1% hypochlorite solution and germinated on glass beads with demineralized water in a growth cabinet at 15–20°C, 8–16 h D/L. Two weeks after germination, the seedlings were transferred to 1-L pots with unsterilized field soil collected from the nature reserve Millingerwaard (51°87′N, 6°01′E). The pots were placed in a greenhouse with conditions of 60% RH, 16 ± 2°C – 21 ± 2°C, 8–16 h D/L in a randomized block design (five blocks). Ten to 20 plants were used of each species. After 8 weeks, defenses in half the plants were induced by placing one *M. brassicae* third instar larva in a clip cages (Ø 8 cm) attached to one leaf of each plant. Leaves of the same age were chosen within each plant species and control plants received clip cages without herbivores. Larvae were weighed before they were placed on the plants. Clip cages were moved to another leaf when the first leaf was almost defoliated. After 5 days of *M. brassicae* feeding, the larvae were removed and weighed again 5 h after removal. Directly after the larvae were removed, all leaves younger than the leaf with the clip cage were harvested and immersed in liquid nitrogen. Leaves were freeze-dried and stored at −80°C until further analysis.

### Untargeted metabolomics using LC-QTOF-MS

Plant samples were analyzed for variation in semipolar metabolite composition using an untargeted accurate mass LC-MS approach, with online absorbance spectra measurements using a photodiode array (PDA) detector, essentially as described in (De Vos et al. 2007). In short, 20 mg DW of frozen plant material was weighed in glass tubes and extracted with 2 mL of 75% methanol in water containing 0.1% formic acid. Samples were sonicated for 15 min at 40 kHz and centrifuged, and then filtered (Captiva 0.45 *μ*m PTFE filter plate; Ansys Technologies, Canonsburg, PA) into 96-well plates with 700-*μ*L glass inserts (Waters, Milford, MA) using a TECAN Genesis Workstation. Extracts (5 *μ*L) were injected using an Alliance 2795 HT instrument (Waters), separated on a Phenomenex Luna C18 (2) column (2.0 × 150 mm, 3 *μ*m particle size) using a 45-min 5–75% acetonitrile gradient in water (both acidified with 0.1% formic acid) and then detected firstly by a photodiode array detector (Waters 2996) at a wavelength range of 220–600 nm and secondly by a Waters-Micromass QTOF Ultima MS with negative electrospray ionization at a mass range of m/z 80–1500. Leucine enkephalin was used as lock mass for online mass calibration.

### Data preprocessing and reduction in the dataset

Metalign software (http://www.metalign.nl) was used to extract and align all accurate mass signals (with signal-to-noise ratio ≥3) from the raw data files. To improve the quality of the dataset, signals present in at least five samples and at least in one an amplitude higher than 100 (about five times the noise value) were subsequently selected, resulting in a dataset of 15,824 mass signals. Finally, the so-called multivariate mass spectra reconstruction strategy (Tikunov et al. 2005) was used to remove data redundancy by both retention time and sample-dependent clustering of signals derived from the same compound, that is, isotopes, adducts, and in-source fragments. This clustering of the 15,824 mass signals revealed 1122 reconstructed metabolites and 896 (5.6%) single, nonclustered, mass signals. From each reconstructed metabolite, the signal intensity of the most intense mass was selected for further statistical analyses. The LC-MS approach mainly detects semipolar nonvolatile secondary metabolites from different biochemical pathways, including phenolics, flavonoids, sesquiterpenes, alkaloids, and saponins, as well as some primary metabolites, such as organic acids and sugars. Both individual mass signals and reconstructed metabolites, based on retention time-dependent clustering of signals over samples (Tikunov et al. 2005), were taken into account.

### Data analysis

Seven quality control samples, consisting of a mixture of the methanol extractions of the 13 plant species used in the experiment, were included in the LC-MS analysis. The error rate of mass signal detection (type II error), calculated as error = 1 – fraction correct^1/*n*^, in these seven control samples was 0.07, which is comparable with other studies using this method (Vorst et al. 2005). Statistical analyses were performed in R 2.11.1 (http://www.R-project.org). Number of total mass signals and total number of metabolites were analyzed with analysis of variance (ANOVA) with origin, herbivory treatment and species nested within origin as fixed factors and the interaction between origin × herbivory treatment included in the model. The number of unique mass signals and unique reconstructed metabolites was not normally distributed and rank-transformed and tested with a multifactorial ANOVA adjusted for ranks (Sokal and Rohlf 1995). Differences within species pairs were tested with separate ANOVAs and significance levels were adjusted for multiple tests with Bonferroni corrections (Sokal and Rohlf 1995). The relative growth of the *M. brassicae* larvae was calculated by end weight divided by begin weight of the larvae. The relative growth data were square-root transformed to meet the assumptions of normal distribution and tested for differences between native and exotic species using ANOVA's with Bonferroni corrections within the congeneric species pairs. Spearman rank correlations were used to test the relation between *Mamestra* relative growth and number of metabolites.

## Results

### Chemical diversity

**Figure 1:**
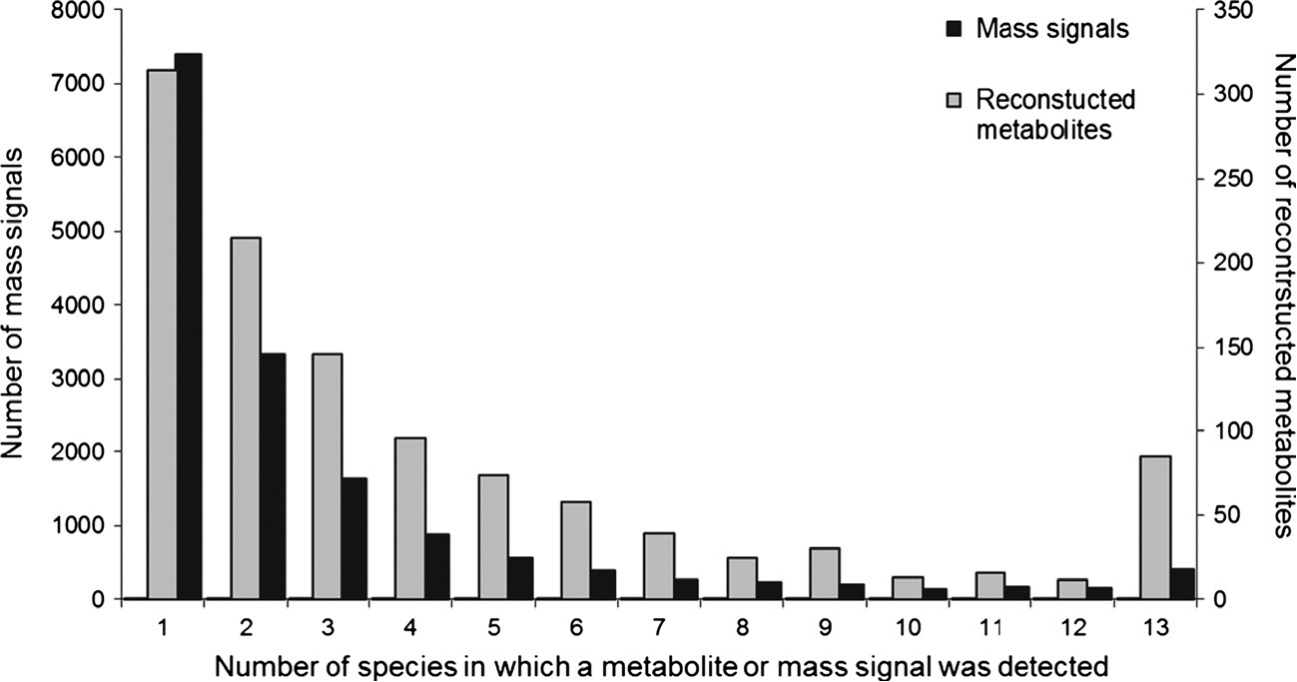
Diversity of metabolites in the 13 analyzed Asteraceae species. Frequency distribution of the mass signals (black bars) and reconstructed metabolites (gray bars).

As a first step to compare the chemical diversity between the Asteraceae species, we analyzed the overlap in metabolomic profiles. Our results indicate a high diversity in secondary chemistry among the tested plant species. Overall, most of the detected mass signals (complete or fragmented metabolites) were species specific (Fig. 1, black bars). Most of the 15,824 individual mass signals, 46.8%, occurred in single plant species. Only 2.6% of all mass signals overlapped and were found in all plant species. Similarity in mass signals between individual plants within a species ranged from 13.3% for *Senecio inaequidens* to 57.5% for *Tr. dubius*. This means that there was considerable variation in chemical profiles within *Se. inaequidens* but variation was much lower within the other species. The similarity in mass signals between all plants within a genus ranged from 2.5% in *Senecio* (three species) to 31% in *Bidens*. The frequency distribution of the reduced dataset, the reconstructed metabolites (cluster data), showed a similar pattern albeit less pronounced (Fig. 1; gray bars), 28% of the 1122 reconstructed metabolites occurred in only a single species while 7.6% of the metabolites were shared among all species. These frequency distributions remained similar when the threshold level was increased to 10 times the noise level, which indicates that the observed distribution was not due to small peaks that are close to the detection limit. One of the metabolites that was present in all plants was chlorogenic acid (mass 353). This phenylpropanoid is commonly found in plants, but is particularly abundant in the Asteraceae (Mølgaard and Ravn 1988).

### Native versus exotic species

**Figure 2:**
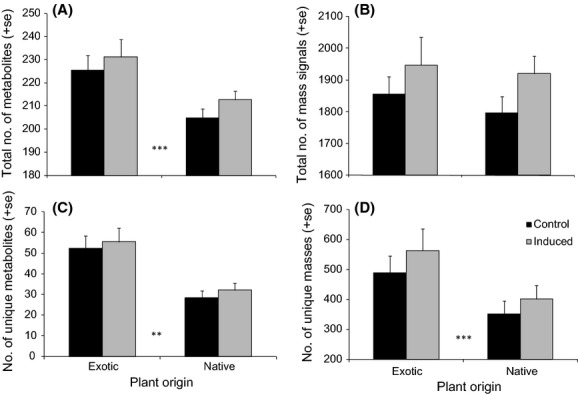
Number of metabolites in native versus invasive plants. Average total number of mass signals (A) and number of reconstructed metabolites (B), number of species-unique masses (C) and species-unique reconstructed metabolites (D) of exotic plants and native congeneric species. Induced plants (gray bars) received herbivory by *Mamestra brassicae* caterpillars. Control plants (black bars) were without herbivory. Plant origin differed significantly for total number of reconstructed metabolites, and unique number of mass signals and reconstructed metabolites (**P < 0.005, ***P < 0.0001, Table 2). Herbivory induced the total number of mass signals and reconstructed metabolites in both native and exotic plants (*P* < 0.05, Table 2). Error bars indicate standard errors of mean.

**Figure 3:**
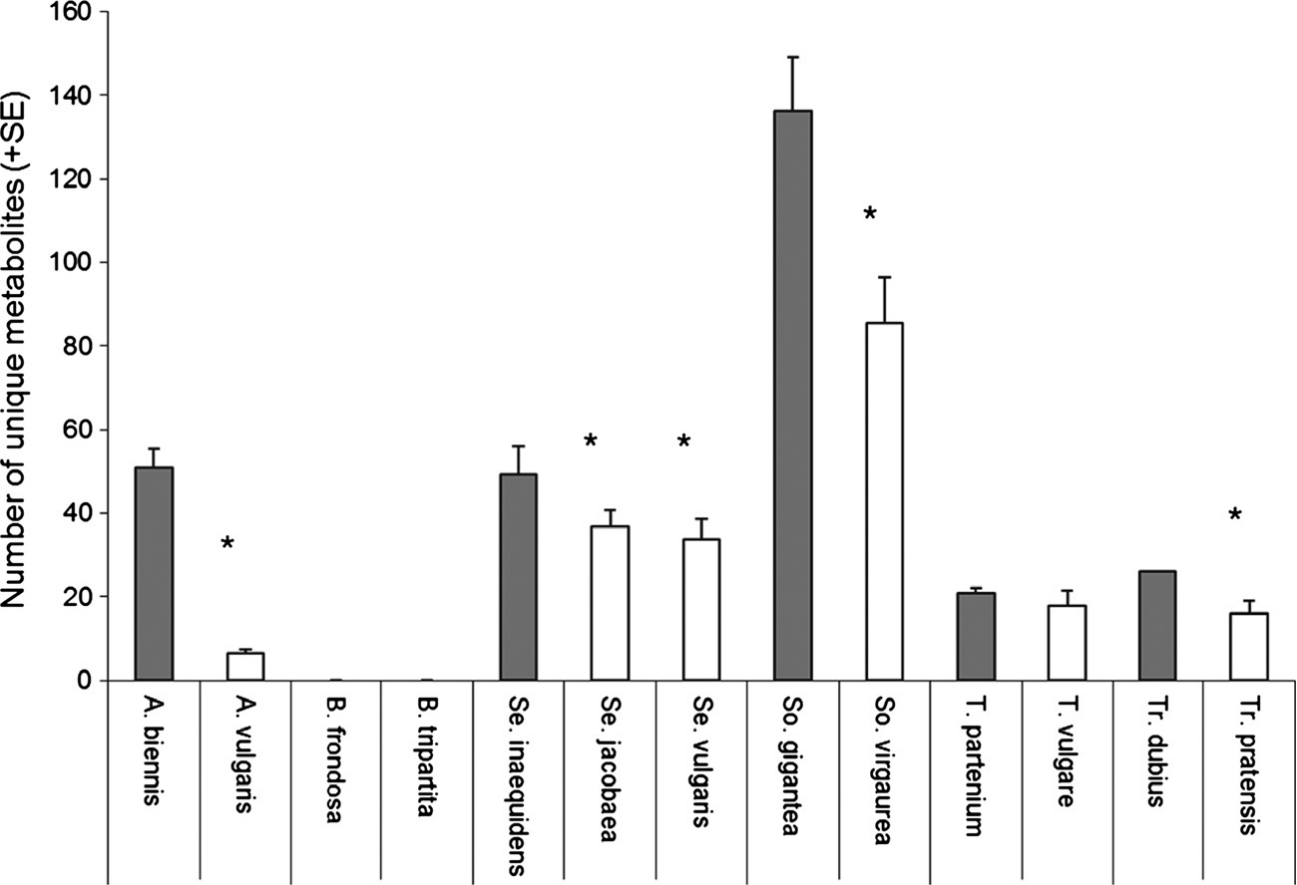
Species-unique metabolites in native versus invasive plants per genus (*Artemisia, Bidens, Senecio, Solidago, Tanacetum, Tragopogon*). Average number (+SE) of unique metabolites per species in native and invasive plants in the control treatment without herbivory. Gray bars indicate exotic species, white bars indicate native species. Asterisks indicate significant differences between exotic and native species within the same genus (ANOVA, all *P* < 0.001).

The range-expanding exotic plant species contained, on average, a higher total number of reconstructed metabolites than their native congeners (Fig. 2A, *P* < 0.0001, Table 2). The total number of mass signals (complete or fragmented metabolites) showed a similar but nonsignificant difference (Fig. 2B, *P* = 0.78). Furthermore, the exotic plants also contained more species-unique metabolites than their native congeners (both mass signals and reconstructed metabolites, Fig. 2C,D, *P* < 0.005, Table 2). The proportion of unique mass signals relative to the total number was also higher in exotic plants (31%) than in natives (24%) (ANOVA on ranks, *H* = 4.51, df = 1, *P* < 0.05). While there was an overall difference between native and exotic species, the differences between genera in number of metabolites were considerable (Table 2, Fig. 3). *Solidago* species, and specifically the exotic *So. gigantea*, accumulated relatively high numbers of unique compounds, more than twice as much as the other species (Fig. 3). On the other hand, the exotic *Bidens frondosa* and its native congener *B. tripartita* shared all metabolites with other species analyzed. When focusing on individual congeneric species pairs, in five out of the seven paired species comparisons, the exotic species possessed more unique metabolites than the native species (Fig. 3).

**Table 2:** Effect of plant origin, species, and herbivory treatment on the number of LC-MS mass signals and reconstructed metabolites in plants

		Mass signals		Reconstructed metabolites	
			
	df	Total	Unique^1^	Total	Unique^1^
Origin	1	0.76	28.52***	69.26***	15.54**
Species within origin	11	74.69***	189.60***	152.68***	203.54***
Treatment	1	10.34**	6.77*	9.54 **	2.11
Origin × Treatment	1	0.14	0.53	0.87	0.17

Table entries are *F* values of multifactorial ANOVA.

*N* = 227.

1 Rank transformed data.

*** *P* < 0.0001,

** *P* < 0.005,

* *P* < 0.05.

### Herbivory

Herbivory increased the number of metabolites in both native and exotic plant species to a similar extent (3.8% and 2.5% increase, respectively, nonsignificant interaction between origin and herbivory treatment), thus the overall pattern of exotic species having more unique chemical compounds remained similar to that in the uninduced plants (Fig. 2 gray bars, Table 2). Interestingly, in three of the four pairs (of the total of seven pairs) where the exotics have more unique metabolites, the relative growth of the *M. brassicae* caterpillars was also significantly lower on the exotic species (*Artemisia, Senecio, Solidago*). Overall, the caterpillars grew 3–10 times faster on the native than on these three exotic species (Table 3). *Tragopogon* was the exception to this pattern, as caterpillars grew significantly faster on the exotic species (Table 3). Overall, there was no direct correlation between the number of unique metabolites and the relative growth rate of the caterpillars [*R*_s_ = 0.01, *P* = 0.94, *N* = 112 (per individual plant and caterpillar) and *R*_s_ = −0.14, *P* = 0.63, *N* = 13 (averages per species)]. Total number of metabolites was also not correlated with caterpillar relative growth (*R*_s_ = −0.01, *P* = 0.91, *N* = 112).

**Table 3:** Relative growth of *Mamestra brassicae* larvae on exotic and native plants. Relative growth was calculated as end weight/begin weight

Genus	Species	Origin	Mean growth (±SE)	N
*Artemisia*	*biennis*	Exotic	0.30 (±0.12)	9
	*vulgaris*	Native	2.96 (±0.67)*	10
*Bidens*	*frondosa*	Exotic	2.70 (±0.85)	5
	*tripartita*	Native	1.36 (±0.63)	4
*Senecio*	*inaequidens*	Exotic	1.33 (±0.23)	10
	*jacobaea*	Native	3.50 (±0.78)+	10
	*vulgaris*	Native	4.30 (±0.67)*	10
*Solidago*	*gigantea*	Exotic	0.72 (±0.31)	10
	*virgaurea*	Native	4.31 (±0.79)*	10
*Tanacetum*	*parthenium*	Exotic	0.85 (±0.20)	9
	*vulgare*	Native	1.63 (±0.47)	9
*Tragopogon*	*dubius*	Exotic	1.44 (±0.31)	6
	*pratensis*	Native	0.35 (±0.10)*	10

Asterisk, significant differences between exotic and native species within congeneric species pairs (ANOVA, significance level after Bonferroni correction for multiple tests *P < 0.008, ^+^*P* = 0.05).

## Discussion

The results of our comparative metabolomics analyses of the different species showed that the successful exotic species had more total and more unique metabolites than native congeners, both in uninduced and herbivore-induced plants. The exotic species were thus overall more chemically diverse than the native species and also more phytochemically unique. Previous studies using more targeted chemical analyses have shown that phytochemical uniqueness may play a role in the invasion of exotic plants (e.g., Callaway and Aschehoug 2000; Cappuccino and Arnason 2006; Kim and Lee 2011; Enge et al. 2012; Svensson et al. 2013). Organisms, for example, herbivores and competitors, in the new or introduced range may not be adapted to the unique compounds that are new to the introduced range (e.g., Callaway and Aschehoug 2000; Callaway et al. 2008; Schaffner et al. 2011; Enge et al. 2012; Svensson et al. 2013). Our results further showed that metabolomes were highly species-specific, with most species containing unique metabolites not found in other species. Both the native and the invasive species were therefore phytochemically unique to some degree, although the proportion of unique metabolites was higher in the exotic plants. Consequently, this indicates that in general any exotic plant species, also noninvasive ones, is likely to have at least some metabolites that are new to organisms in the introduced range. We cannot be absolutely certain that the unique compounds found here are not present in any other species as we included only one or two native sister species in our study. Nonetheless, the high proportion of unique metabolites of the successful exotic plants studied here suggests that high chemical diversity and high phytochemical uniqueness may be indicative of biological invasion potential. High chemical diversity and greater chemical uniqueness can be beneficial in several ways. High diversity of plant secondary metabolites can lead to higher resistance to, for instance, herbivory by impeding counter-adaptations by (native) herbivores and by making the plant more toxic if compounds act synergistically. Furthermore, plants with high chemical diversity may be resistant to a wider range of antagonists if individual metabolites act specifically against different organisms (Berenbaum 1985; Jones and Firn 1991; Fritz and Simms 1992). Possibly most importantly, the high proportion of unique chemicals in exotic plant species could increase the chance of having a potent compound or combinations of compounds to which native species in the introduced range are not adapted yet.

In our metabolomic fingerprinting approach, we did not attempt to identify all of the metabolites because of the large number of unknown metabolites that were detected (95%). Therefore, we cannot distinguish whether the exotic plants in this study contained completely different classes of compounds compared with native species, or produced compounds that were structurally related to compounds present in the native species. Although it may be more likely that organisms in the introduced range of an exotic species are not adapted to a metabolite from a class of compounds that is completely absent in the introduced range, small modifications of structurally related compounds may already require new adaptations as they can have different modes of action (Macel et al. 2005; van Leur et al. 2008). With the method we used, we mostly analyzed plant secondary metabolites, which generally have a function in the plant's interactions with its biotic and abiotic environment (Fritz and Simms 1992). In how far the metabolites analyzed in this study are used as defenses or offense (novel weapons) in the new range we cannot say.

We included the exotic invaders from other continents and the exotic range expanders in our study. In both groups of exotics, exotic plants had more unique metabolites than the natives in two out of the three congeneric species pairs (Fig. 3). Invasion processes from exotic species from other continents may be different from range-expanding exotics, such as only partial enemy release and continuing gene flow with source populations in the native range (Morrien et al. 2010). Nonetheless, high levels of chemical diversity and chemical uniqueness in individual plants could be related to successful spread and/or invasion of exotic species, independent of their origin. Furthermore, plants from lower latitudes are expected to have stronger defenses against herbivory due to the greater herbivore pressure at lower latitudes compared with higher latitudes (Bolser and Hay 1996; Pearse and Hipp 2012). Plants that are shifting to higher latitude areas could therefore include highly defended plants. It is also possible that selection during range expansion or invasion favors plants with a higher chemical diversity and chemical uniqueness.

It would be interesting to see whether the results that we found here would be similar on other continents as well. Some of the native species in our study are invasive elsewhere, such as *Se. jacobaea*, *Se. vulgaris*, *T. vulgare*, and *A. vulgaris*. In the introduced range of an exotic species, intraspecific hybridization (admixture) can occur between populations that were isolated from each other in the native range. Admixture is thought to play an important role in biological invasions (Ellstrand and Schierenbeck 2006; Verhoeven et al. 2011). Benefits of admixture include an increase in standing genetic variation, the formation of novel genotypes, and lift of inbreeding load. A recent study showed that outbreeding increases the number of phenolic compounds in plants (Campbell et al. 2013). If outbreeding in general increases, the number of defense compounds in plants and intraspecific hybridization has occurred in the exotic species, then it is possible that successful invasive admixed genotypes in the introduced range of a species could have a higher number of defense compounds than plants in their native range. This would be an additional explanation for the higher numbers of metabolites in the exotic plants.

The performance of the native generalist herbivore *M. brassicae* was significantly lower on three of the six exotic species when compared with their native congeners (*Artemisia*, *Senecio*, *Solidago*). This suggests that some, but not all, of the exotic species in our study were better defended than native species against this native herbivore. The three exotic species on which *M. brassicae* performed poorly also contained significantly higher number of metabolites than the native sister plants. However, we did not find a direct linear correlation between herbivore performance and number of metabolites among all the species. Possibly only a few metabolites or a combination of active compounds are responsible for the low performance of this particular herbivore (van Leur et al. 2008). Larval performance on the exotic range-expanding *Tragopogon* species was higher compared with performance on the native *Tragopogon*, even though the exotic species contained a higher number of metabolites. We expected that generally herbivore performance on the exotic plants would be lower, but here we found that it does not hold for all the exotic species we tested. Indeed there is quite some variation in the results obtained with manipulative herbivore experiments in which native and exotic congeners are compared. For example, it was shown that generalist snails fed more on the exotic *Se. inaequidens* than on the native *Se. vulgaris* (Cano et al. 2009). In an early study on range-expanding exotic plants, generalist locusts were performing worse on the exotics, while generalist aphids performed equally well on both exotics and natives (Engelkes et al. 2008). The palatability of exotic species thus also depends on which native generalist herbivore species is tested.

In conclusion, our untargeted metabolomics study showed that successful exotic plant species had a higher diversity of metabolites and more unique metabolites compared with congeneric native species. This pattern was found for classic invaders from other continents as well as for plants that are currently successfully expanding their range on the same continent. In addition to one single highly potent novel compound, high chemical diversity and phytochemical uniqueness of many compounds may thus also be indicative of plant species invasiveness. Furthermore, combinations of compounds acting in synchrony are likely to be important. The exact function of the high chemical diversity and uniqueness in exotic plants and its role in plant invasions still needs further testing. Whether this high diversity is due to postintroduction evolution or is a pre-existing trait of invasive exotic plants also remains to be elucidated.
